# Different Roles of COMT and HTR2A Genotypes in Working Memory Subprocesses

**DOI:** 10.1371/journal.pone.0126511

**Published:** 2015-05-14

**Authors:** Hirohito M. Kondo, Michio Nomura, Makio Kashino

**Affiliations:** 1 Human Information Science Laboratory, NTT Communication Science Laboratories, NTT Corporation, Atsugi, Kanagawa 243–0198, Japan; 2 Department of Child Development, United Graduate School of Child Development, Osaka University, Suita, Osaka 565–0871, Japan; 3 Division of Cognitive Psychology in Education, Graduate School of Education, Kyoto University, Kyoto 606–8501, Japan; 4 Department of Information Processing, Interdisciplinary Graduate School of Science and Engineering, Tokyo Institute of Technology, Yokohama, Kanagawa 226–8503, Japan; UTHSCSH, UNITED STATES

## Abstract

Working memory is linked to the functions of the frontal areas, in which neural activity is mediated by dopaminergic and serotonergic tones. However, there is no consensus regarding how the dopaminergic and serotonergic systems influence working memory subprocesses. The present study used an imaging genetics approach to examine the interaction between neurochemical functions and working memory performance. We focused on functional polymorphisms of the catechol-*O*-methyltransferase (COMT) Val^158^Met and serotonin 2A receptor (HTR2A) -1438G/A genes, and devised a delayed recognition task to isolate the encoding, retention, and retrieval processes for visual information. The COMT genotypes affected recognition accuracy, whereas the HTR2A genotypes were associated with recognition response times. Activations specifically related to working memory were found in the right frontal and parietal areas, such as the middle frontal gyrus (MFG), inferior frontal gyrus (IFG), anterior cingulate cortex (ACC), and inferior parietal lobule (IPL). MFG and ACC/IPL activations were sensitive to differences between the COMT genotypes and between the HTR2A genotypes, respectively. Structural equation modeling demonstrated that stronger connectivity in the ACC-MFG and ACC-IFG networks is related to better task performance. The behavioral and fMRI results suggest that the dopaminergic and serotonergic systems play different roles in the working memory subprocesses and modulate closer cooperation between lateral and medial frontal activations.

## Introduction

Working memory (WM) refers to systems involved in holding and manipulating representations in the mind, and it supports cognitive abilities, such as reading comprehension, spatial thinking, and associative learning [1, 2]. WM processes are regulated by the dopaminergic system, which enhances the neural signal-to-noise ratio in the frontal cortex [3, 4]. Catechol-*O*-methyltransferase (COMT) is involved in the degradation of synaptic catecholamines: COMT containing Met is one-fourth as active as that containing Val. The less efficient metabolism leads to higher levels of dopamine remaining in and around the synapses [5, 6]. It has been found that Val/Val homozygotes show greater frontal activations during WM tasks, such as the *n*-back task, than Met carriers [7–9]. However, recent studies have demonstrated an opposing pattern of activations [10, 11]. Thus, there is still some controversy about how the COMT Val^158^Met genotypes affect WM-related brain activity.

It is known that serotonin modulates emotional behaviors and its dysfunction causes depression, mood disorders, and impulsive behavior [12–14]. The serotonergic system, like the dopaminergic system, plays an important role in human cognition. It has been reported that the polymorphism of serotonin 2A receptor (HTR2A) His452Try is associated with the delayed recall accuracy of a verbal memory task [15]. However, subsequent results have been mixed: the administration of psilocybin (an HTR2A agonist) has no effect on visual working memory [16], and the HTR2A T102C polymorphism is linked to perseverative errors in the Wisconsin Card Sorting Test [17]. In addition, there is evidence that frontal activations during the *n*-back task are different for different genotype groups of tryptophan hydroxylase 2 (a regulator of serotonin synthesis), but, despite the differences, task performance remains unchanged [18]. The present study focused on the relationship between the HTR2A -1438G/A polymorphism and WM-related brain activity. The -1438A allele enhances promoter activity (i.e., gene expression of HTR2A) more than the -1438G allele does [19]. HTR2A is abundant on the apical dendrites of pyramidal cells in the frontal cortex [20] and the activity of HTR2A increases cortical activations via glutamatergic excitatory postsynaptic potentials [21]. Thus, we can expect brain activity during a WM task to be greater for A carriers than for G carriers.

**Fig 1:**
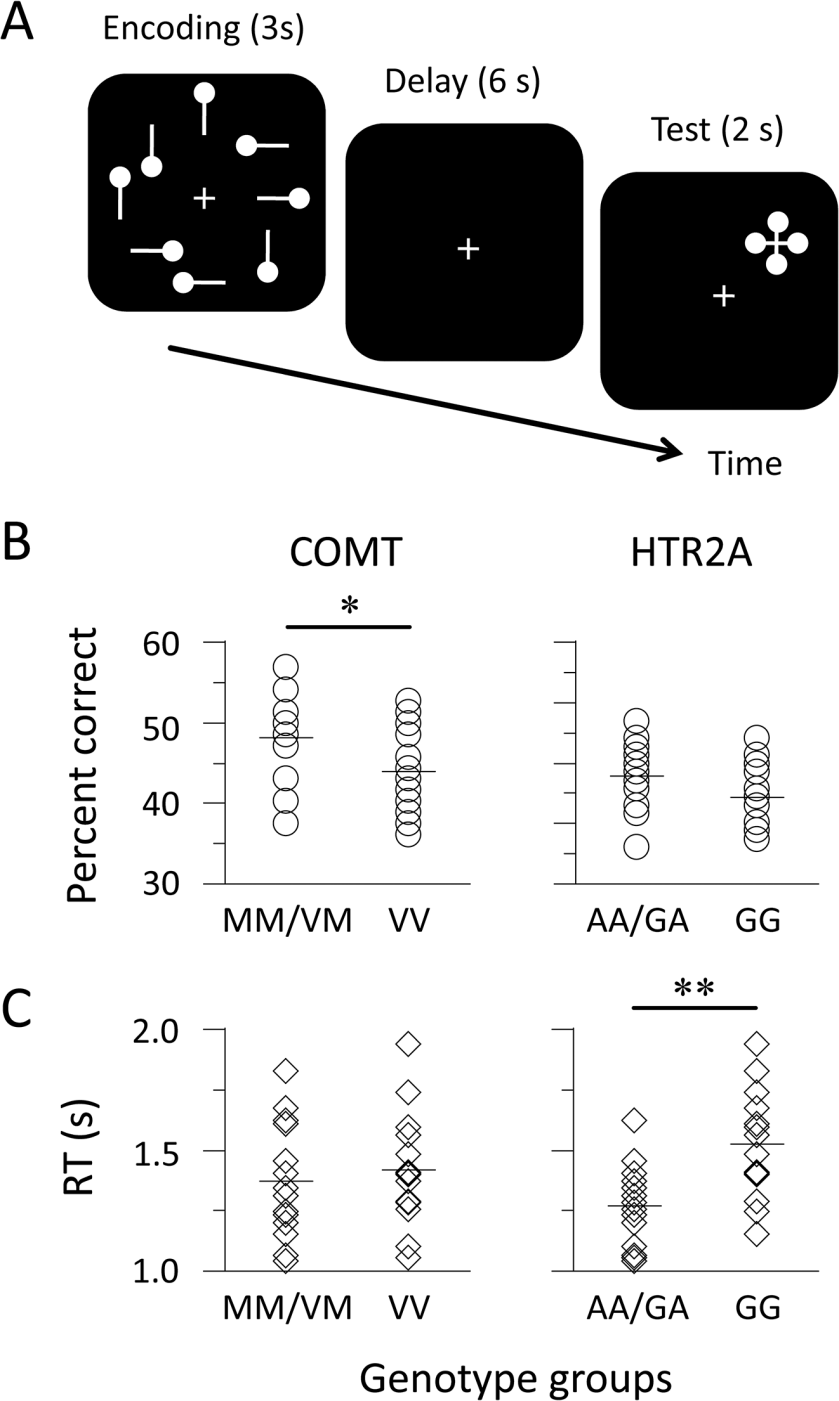
Schematic representation of stimuli and task performance. (A) Participants memorized a sample display during the encoding phase and retained the configuration of circles during the delay phase. When a probe appeared during the test phase, they indicated the position of the target circle from four possible positions by pressing a button. (B and C) Circles and diamonds indicate the percentage of correct responses and the median of response times (RTs) for each individual, respectively. Horizontal bars represent the mean of the genotype groups. ** *p* < 0.01, * *p* < 0.05, *N* = 14 for each group.

The discrepancies among the results across previous studies may reflect different subprocesses within tasks, which have differential sensitivity to neurotransmitter activity [9, 22]. The *n*-back task has been frequently employed as a WM measure, but it includes subprocesses for retention, matching, and updating, which are inseparable. In addition, the phonological component of WM systems has been mainly investigated, but executive control seems to be required more for visual tasks than for verbal tasks [23, 24]. We developed a simple task that comprises separate processes for the encoding, maintenance, and retrieval of visual information (Fig 1A) and identified brain activity during the task phases using functional magnetic resonance imaging (fMRI). Another possibility is that the effective connectivity between brain activations, rather than the signal strength of the activations, is closely linked to WM performance [25–27]. It has been shown that individual differences in visual WM performance strongly depend on the functions of the fronto-parietal networks, particularly the right hemisphere [27, 28]. Dopaminergic neurons are projected from the brainstem to the frontal and limbic areas [29]. The catabolic flux of synaptic dopamine through the COMT pathway is characteristic of the frontal cortex [30]. Although the projection of serotonergic neurons spreads widely throughout the whole brain [29], the function of the HTR2A is pronounced in the frontal cortex [31]. Taking these findings into account, we hypothesized that the frontal and parietal areas are sensitive to differences in brain activity of genotype groups.

This study was designed to look at two factors: genotype group and task phase. We first assessed the degree to which variations in the COMT and HTR2A genotypes contribute to WM performance. For each task phase, we used a subtraction analysis to compare the strength of brain activity between genotype groups. We constructed interregional networks for each group and specified the effective connectivity between brain areas.

## Materials and Methods

### Ethics Statement and Participants

All procedures reported in this study were approved by the Ethics and Safety Committees of NTT Communication Science Laboratories and ATR Institute International (approval numbers: H18-008 and 07–001). All participants gave written informed consent after the procedures had been fully explained to them. Data are available from the Ethics Committee of NTT Communication Science Laboratories for researchers who meet the criteria for access to confidential data. Access is limited to maintain privacy.

An initial pool of 90 right-handed Japanese (45 males and 45 females; mean age 22.6 years, range 20–29 years) was genotyped for screening [32, 33]. They were in good health without any previous history of neurological or psychiatric disorders and had normal or corrected-to-normal visual acuity. Venous blood samples were collected from all participants and genomic DNA was extracted. The polymorphisms of the COMT Val^158^Met (dbSNP accession: rs4680) and HTR2A -1438G/A (rs6311) genes were genotyped. Polymerase chain reactions (PCRs) were carried out using PrimeSTAR HS DNA polymerase in a total volume of 20 μl containing 60 ng genomic DNA, 2.5 mM of each dNTP, 5 mM of 5× PCR buffer, and 8 μM of each primer. The single nucleotide polymorphisms were amplified with the primers of COMT (fw: 5’ CAC CTG TGC TCA CCT CTC CT 3’; rev: 5’ GGG TTT TCA GTG AAC GTG GT 3’) and HTR2A (fw: 5’ AAC CAA CTT ATT TCC TAC CAC 3’; rev: 5’ TAA GCT GCA AGG TAG CAA CAG 3’). Initial denaturation at 98°C for 2 min was followed by 35 cycles of denaturation at 98°C for 10 s, primer annealing at 60°C for 5 s, and primer extension at 72°C for 60 s. PCR products were genotyped with an Applied Biosystems 3730 DNA Analyzer with a BigDye Terminator Cycle Sequencing Kit. The polymorphisms were distributed according to the Hardy-Weinberg equilibrium: 16 Met/Met, 38 Val/Met, and 36 Val/Val individuals of the COMT genotype (*χ* ^2^_(1)_ = 1.12, *p* > 0.29); 24 A/A, 43 G/A, and 23 G/G individuals of the HTR2A genotype (*χ* ^2^_(1)_ = 0.18, *p* > 0.67).

All participants performed six types of paper-and-pencil tests that were chosen from the Kyoto University SX15 Intelligence Test [34]. The Word Selection and Sentence Completion Tests were used to assess the verbal abilities of the participants, and the Mental Rotation and Paper Unfolding Tests were chosen to investigate their abilities with respect to mental imagery operations. The other two subtests, the Operation Exchange and Calculation Method Tests, were used to check their arithmetic performance. The number of correct answers for each subtest was transformed into *z* scores (mean = 50) for each individual. The scores were added up to compute composite scores for each domain. The reliability estimates of the subtests were calculated by adjusting split-half correlations with the Spearman-Brown prophecy formula. The estimates generally reached a satisfactory level (0.68 to 0.84 range).

Twenty-eight of the 90 participants were recruited for the fMRI experiment. To minimize confounding factors, we carefully matched genotype groups in terms of gender, age, years of education, handedness scores (as measured with the Edinburgh Handedness Inventory), and composite scores of intelligence subtests (Table 1). We categorized the participants into two equal groups because of the small number of Met/Met individuals: MM/VM (4 Met/Met and 10 Val/Met individuals) and VV (14 Val/Val individuals) groups. Similarly, we divided the same participants into AA/GA (7 A/A and 7 G/A individuals) and GG (14 G/G individuals) groups. The numbers of AA/GA and GG participants were balanced in each COMT genotype group; each HTR2A genotype group comprised the same numbers of MM/VM and VV participants.

**Table 1:** Demographic characteristics of COMT and HTR2A genotype groups.

Measure	COMT genotypes		HTR2A genotypes	
	MM/VM	VV	AA/GA	GG
Gender (male/female)	6/8	6/8	6/8	6/8
Age	22.9 (2.3)	23.7 (2.4)	22.9 (2.1)	23.7 (2.6)
Education	16.4 (1.5)	16.8 (1.1)	16.4 (1.2)	16.9 (1.3)
Handedness	93.2 (8.9)	94.6 (7.5)	96.8 (7.2)	91.1 (8.1)
Psychometric test				
Verbal score	101.3 (14.0)	100.1 (9.8)	101.1 (15.7)	100.3 (6.8)
Spatial score	102.6 (15.2)	100.7 (16.3)	101.2 (16.2)	102.1 (16.4)
Arithmetic score	94.8 (15.0)	91.9 (16.2)	94.3 (14.8)	92.4 (16.4)

The means and standard deviations (in parentheses) are shown. Positive values of handedness scores indicate strongly right-handed. The intelligence test scores are normalized (mean score = 100) by using data for 90 individuals.

### WM Task

At the beginning of each trial, crosshairs were displayed for fixation. An encoding display was presented for 3 s, a blank screen for 6 s, and a test display for 2 s (Fig 1A). The duration of the trial was fixed, but the inter-trial interval was temporally jittered (3 to 5 s range). On each encoding display, eight white bars on a black background were presented around the crosshairs (visual angle 6.0°). Vertical or horizontal bars were randomly used for each presentation. A small circle was located on either side of each bar. Participants were instructed to memorize the configuration of the circles as a visual image during the encoding phase and keep track of it during the delay phase. During the test phase, a cross-shaped probe appeared with four circles indicating four possible positions so that the chance of correctly guessing the answer was 25%. We used a response box with four buttons whose positions formed a diamond shape corresponding to the position of the target circle in the encoding display. Participants were instructed to press a button with their right thumb. Their responses were recorded via a nonmagnetic response device (Current Designs, Philadelphia, PA, USA).

Participants completed six runs (6 min 8 s for each) and performed a total of 144 trials. Before an actual run, they completed one practice run to familiarize themselves with the task. Stimuli were generated on a PC and projected onto a translucent screen placed outside the bore of the magnet. Participants viewed stimuli via a mirror system attached to a head coil and pressed a response button with their right thumb. Presentation software (Neurobehavioral Systems, Berkeley, CA, USA) was used to synchronize the timing of the stimuli presentation with that of the scan sequence.

### Image Data Acquisition

Images were acquired with a 1.5-T MRI scanner (Shimadzu-Marconi, Kyoto, Japan) with a standard head coil. Head motion was minimized with comfortable padding around the participant’s head. Functional images sensitive to blood oxygen level dependent signals were obtained with a single-shot echo-planar imaging sequence (2-s repetition time, 48-ms echo time, 80° flip angle, 64 × 64 at 3 mm in-plane resolution, 6-mm thickness, 184 volumes for each session). The whole brain, except for the orbitofrontal area and the lower part of the cerebellum, was covered with 20 contiguous oblique transverse slices. After the experimental runs, anatomical images were obtained with a standard T1-weighted pulse sequence (isotropic voxel size of 1 mm^3^).

### Image Data Analyses

Imaging data were reprocessed with SPM8 (Wellcome Trust Centre for Neuroimaging, London, UK) implemented in Matlab (MathWorks, Natick, MA, USA). The four initial images of each run were discarded from the analysis to achieve steady-state equilibrium between radio-frequency pulsing and relaxation. The images were calibrated to correct the slice acquisition timing and realigned to correct the head movement. The movement on the *x*, *y*, and *z* axes was less than 1 mm within runs. The images were co-registered to the anatomical images, normalized to the T1 template of the Montreal Neurological Institute (MNI), and spatially smoothed with a 9-mm Gaussian full-width at half-maximum filter.

After preprocessing, we performed a first-level analysis for each individual. The design matrix was created to identify genotype- and WM-related activations. The 3-s encoding, 3-s delay, and 2-s test phases were modeled using three box-car regressors with a canonical hemodynamic response function. The middle 3 s of the delay phase was set as a regressor to prevent retention-related activity from contaminating encoding- and retrieval-related activity. This resulted in at least a 4-s separation between the onset of task phases, which allows the analysis to statistically resolve temporally neighboring signals [35]. The head motion realignment parameters were also included as a regressor. The data were high-pass filtered with a cut-off at 128 s to remove baseline drift and whitened using a first-order autoregressive model to control the temporal autocorrelation. Subject-specific activation maps were used for a second-level analysis (one-sample *t* tests; *N* = 28). In the subsequent analysis, the statistical significance was set at a threshold of uncorrected *p* < 0.001 and at a cluster size of *k* > 30. A subtraction analysis was performed to detect differences between the brain activations of the MM/VM and VV groups and of the AA/GA and GG groups (two-sample *t* tests; *N* = 14 for each).

To compare the connectivity of brain activations between the genotype groups, we constructed interregional networks using structural equation modeling. The connectivity analysis was performed for each task phase. On the basis of the results of the genotype group comparisons, the following areas were chosen as regions of interest (ROIs): the middle frontal gyrus (MFG, Brodmann area 46), inferior frontal gyrus (IFG, BA 45), anterior cingulate cortex (ACC, BA 32), and inferior parietal lobule (IPL, BA 40) in the right hemisphere. The structural model consisted of four ROIs and their connections with each other. We focused on the encoding and delay activations, determined the ROIs of each individual anatomically, and extracted signal changes from the local maxima of the ROIs (see Table 2). The peak amplitudes of the signal changes were specified for each task phase. Following procedures used in previous studies [36–38], we concatenated peak amplitude data to check for a general tendency in brain activity (sample size, *N* = 2016; 144 trials × 14 participants). Models were computed by minimizing the difference between observed and predicted covariances of peak amplitudes. To assess the models, we chose the Akaike information criterion (AIC) and the comparative fit index (CFI) because the *χ* ^2^ statistic easily reaches a significant level in the analysis of large data sets. The AIC is an absolute fit index and a lower AIC value reflects a better-fitting model. The CFI, a relative fit index, quantifies the extent to which the evaluated model is better than an independence model, in which correlations between observed variables are set at zero. The parameter in the model was represented as a path coefficient between ROIs, corresponding to an estimate of effective connectivity. *Z* tests were employed to examine whether path coefficients differed between genotype groups. Finally, a correlation analysis was carried out to investigate the brain-behavior relationship. We produced structural models for each individual, identified path coefficients between ROIs, and estimated correlations between the coefficients and task performance. These analyses were performed with IBM SPSS Statistics and Amos (version 22).

**Table 2:** Activated areas related to working memory subprocesses (*N* = 28).

Region	BA	Encoding					Delay					Test				
		*x*	*y*	*z*	*t* value	*k*	*x*	*y*	*z*	*t* value	*k*	*x*	*y*	*z*	*t* value	*k*
MFG	L10	38	56	6	7.29	296										
	R10	-34	52	8	6.16	210	38	56	-2	7.00	428	44	56	-4	7.80	204
	L46/9	-44	28	26	5.21	71	-42	26	30	5.19	309					
	R46/9	44	44	24	4.22	170	44	38	24	4.08	64					
IFG	L45/44						-46	0	6	9.82	1299					
	R45/44	46	18	10	4.24	62	52	22	4	9.28	1484					
ACC	L32	-12	28	24	4.67	59	-2	38	24	5.28	228					
	R32	10	28	30	3.65	47	8	36	26	6.17	191					
IPL	L40	-42	-40	44	12.61	379	-54	-36	36	9.73	783	-48	-54	42	6.98	203
	R40	44	-38	42	10.33	448	56	-40	38	11.51	679	46	-38	42	5.44	281
SPL	L7	-16	-62	62	10.63	256										
	R7	10	-70	56	8.44	357										
LING	L18											-8	-78	-8	6.75	342
	R18											12	-76	-6	8.29	463

All voxels are significant at the threshold of uncorrected *p* < 0.001 and at a cluster size of *k* > 30. MFG, middle frontal gyrus; IFG, inferior frontal gyrus; ACC, anterior cingulate cortex; IPL, inferior parietal lobule; SPL, superior parietal lobule; LING, lingual gyrus; BA, Brodmann area; L, left; R, right.

## Results

### WM Performance

The accuracy and response time (RT) for recognition were used as measures of WM performance. We performed a statistical analysis using raw data because the variables followed a normal distribution (Kolmogorov-Smirnov tests, *p* > 0.20). For the COMT genotypes, the accuracy (mean ± standard error) was better for the MM/VM group (48.2 ± 1.5%) than for the VV group (44.0 ± 1.4%): *t*_(26)_ = 2.07, *d* = 0.81, *p* = 0.049 (Fig 1B). However, for the HTR2A genotypes, there was no difference in performance between the AA/GA and GG groups: 47.9 ± 1.5% and 44.4 ± 1.5%; *t*_(26)_ = 1.67, *d* = 0.66, *p* > 0.11. A regression analysis demonstrated that the COMT and HTR2A genotypes accounted for the 20.8% variance in recognition accuracy: adjusted *R* ^2^ = 14.5%, *F*_(2, 25)_ = 3.39, *p* = 0.050. A post-hoc power analysis showed that the statistical power (i.e., (1 - *β*) probability) was 0.537. We compared the genotypes in terms of median RTs (Fig 1C). There was no difference between the RTs of the MM/VM and VV groups (1371 ± 64 ms and 1421 ± 63 ms): *t*_(26)_ = 0.56, *d* = 0.20, *p* > 0.50. In contrast, the RTs were faster in the AA/GA group (1268 ± 45 ms) than in the GG group (1524 ± 60 ms): *t*_(26)_ = 3.40, *d* = 1.34, *p* = 0.002. The statistical power for the RTs was 0.552. These results indicate that the COMT and HTR2A genotypes have different effects on the performance of a visual WM task.

### WM- and Genotype-Related Activations

**Fig 2:**
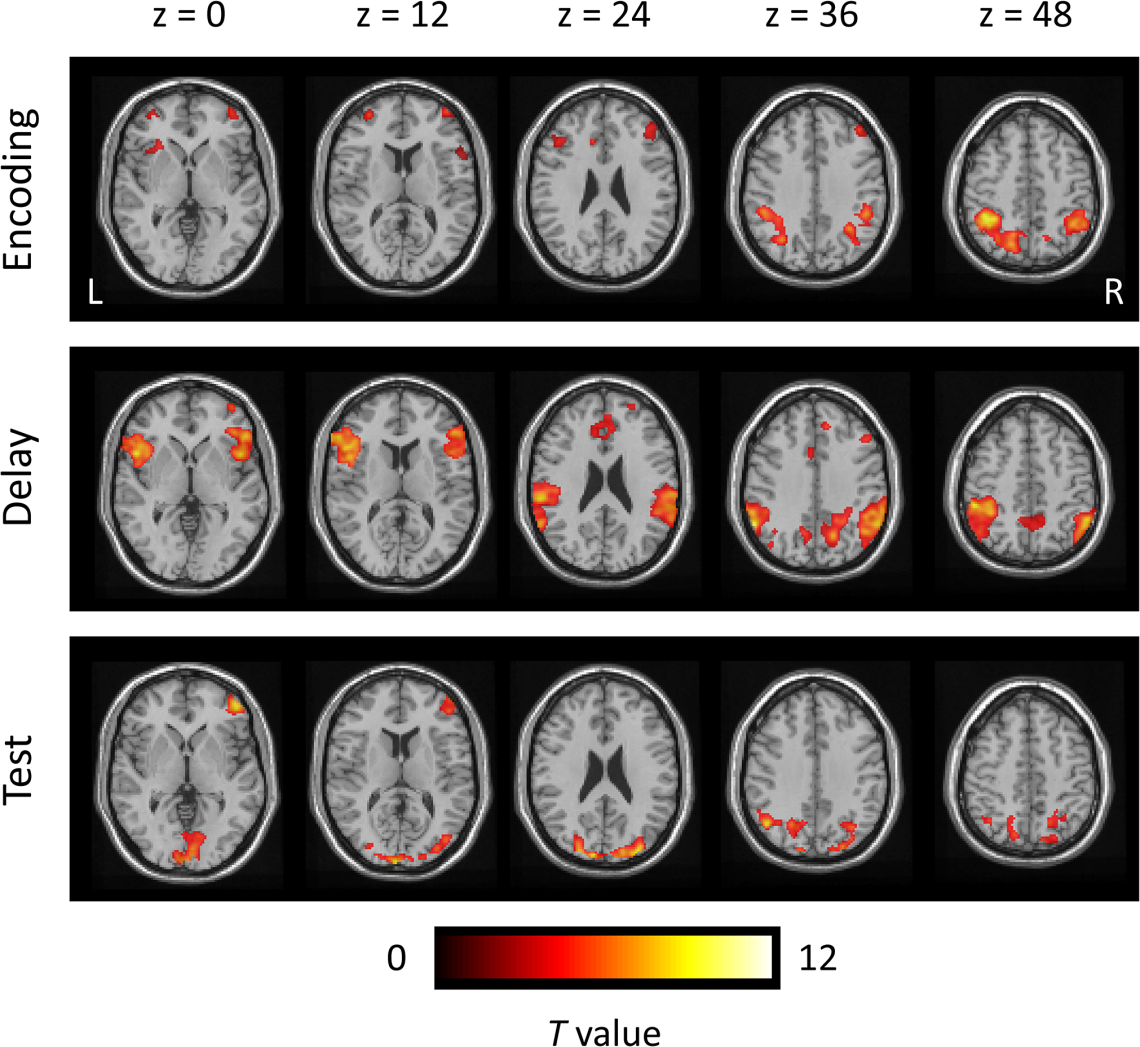
Average activations related to WM subprocesses. Color-coded *t*-statistic maps are superimposed on a transverse slice of grayscale anatomical images. The activations are timelocked with different events for each trial: 0–3 s = encoding; 4.5–7.5 s = delay; 9–11 s = test. The numbers above the top panel refer to *z* coordinates in the MNI space. All activations are significant at the threshold of uncorrected *p* < 0.001 (*t* > 3.24). R, right; L, left.

We first identified average activations (*N* = 28) related to WM subprocesses (Table 2). Activation areas time-locked to the encoding, delay, and test phases are shown in Fig 2. The MFG and IPL were activated throughout all of the phases. In addition, ACC and IPL activities were found during the encoding phase, whereas IFG and ACC activities were specified during the delay phase. A subtraction analysis was performed to compare the extent and intensity of activations between the genotype groups (*N* = 14 for each) (Table 3). Although distributed frontal and parietal areas were activated throughout a trial, the contrasts between the genotype groups produced localized activations, particularly of the right hemisphere. The contrast of the VV-minus-MM/VM groups produced MFG activity during the encoding phase. However, there was no significant activation in the contrast of the MM/VM-minus-VV groups. In the contrast of the AA/GA-minus-GG groups, the ACC and IPL were activated during the delay phase. However, there was no significant activation in the contrast of the GG-minus-AA/GA groups. These results suggest that the involvement of the dopaminergic and serotonergic systems in brain activity varies with the WM subprocesses.

**Table 3:** Activated areas in the contrasts of genotype groups (*N* = 14 for each).

Region	BA	Encoding					Delay				
		*x*	*y*	*z*	*t* value	*k*	*x*	*y*	*z*	*t* value	*K*
VV minus MM/VM											
MFG	R46	44	36	22	5.00	57					
AA/GA minus GG											
ACC	R32						14	24	40	4.46	95
IPL	R40						54	-36	42	4.35	54

See the Table 2 legend for statistical significance and abbreviations.

### Interregional Network Models for Genotype Groups

**Fig 3:**
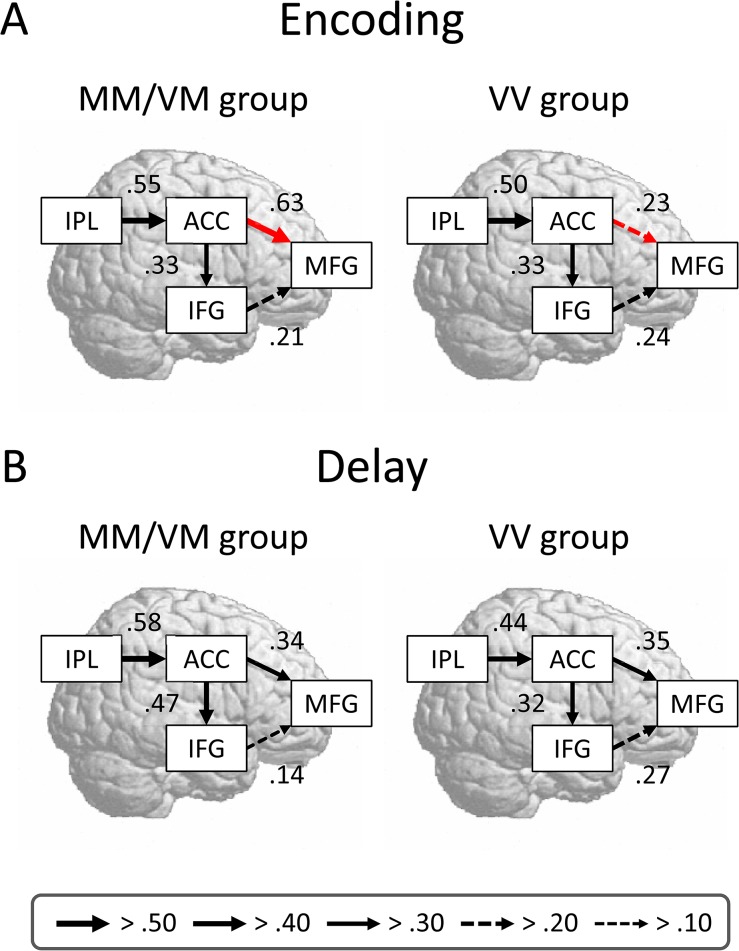
Comparisons of path diagrams of the COMT genotype groups. (A and B) Interregion network models are derived from the encoding and delay activation data, respectively. The signal changes are extracted from local maxima of brain activations for each phase (see Table 2). The peak amplitudes of signal changes are entered as the variables. The values alongside the arrows represent the path coefficients, corresponding to the estimates of effective connectivity. The red arrows indicate significant differences between the coefficients of the genotype groups (*p* < 0.05).

**Fig 4:**
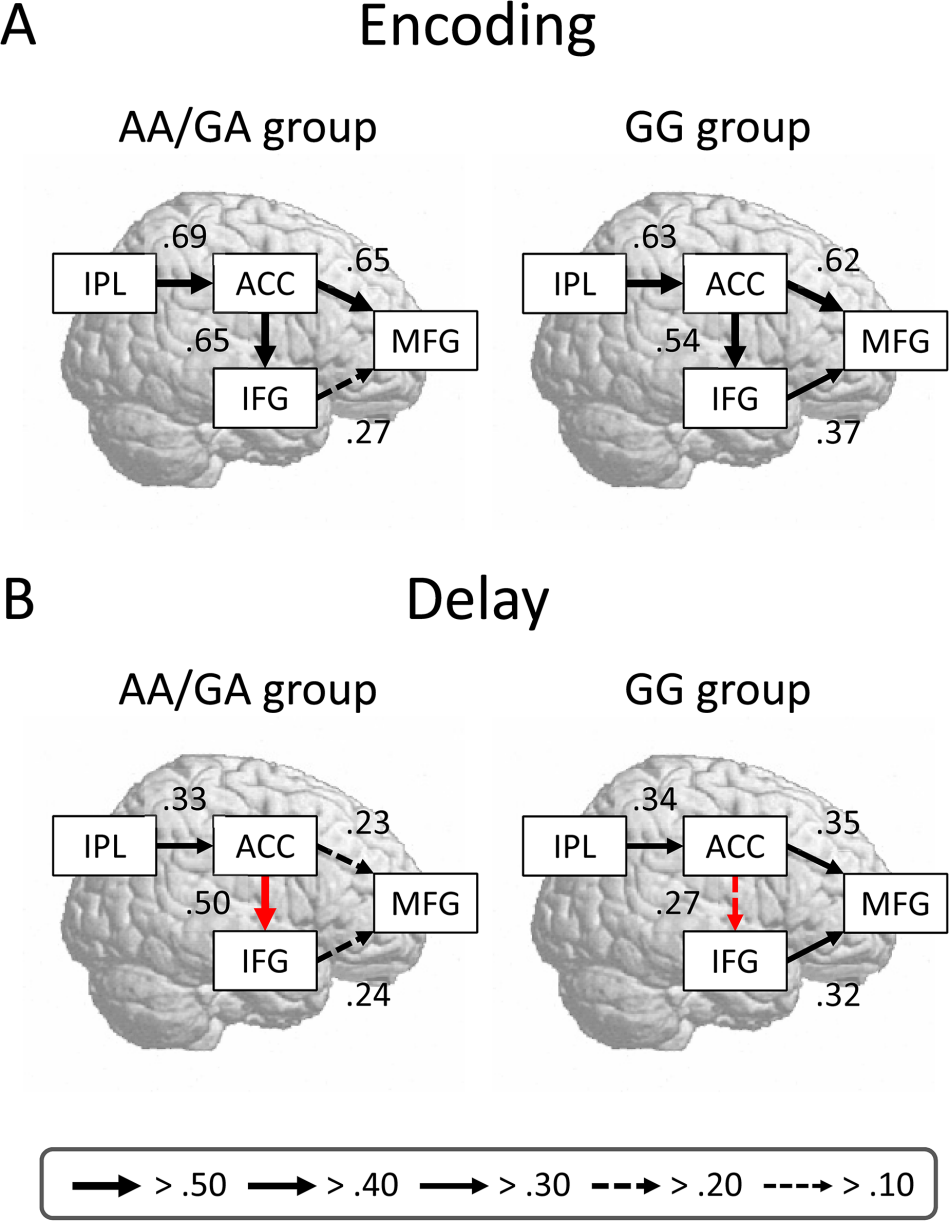
Comparisons of path diagrams of the HTR2A genotype groups. See the Fig 3 legend for a detailed description.

We constructed interregional networks for each genotype group to examine the effective connectivity between brain activations. On the basis of the contrasts between the genotype groups, we focused on the encoding and delay activations. Using structural equation modeling, we assessed the goodness of fit for the models and specified estimates of effective connectivity between ROIs. In all the models, the values of the fit indices generally reached a satisfactory level (Table 4). The models for the MM/VM and VV groups are shown in Fig 3. There was a difference between the effective connectivities of the COMT genotypes during the encoding phase, but not during the delay phase. The estimate of effective connectivity from the ACC to the MFG was greater for the MM/VM group (0.63) than for the VV group (0.23): *z* = 4.26, *p* < 0.001. The models for the AA/GA and GG groups are shown in Fig 4. The difference between the effective connectivities of the HTR2A genotypes was found during the delay phase, but was not during the encoding phase. The estimate of effective connectivity from the ACC to the IFG was greater for the AA/GA group (0.50) than for the GG group (0.27): *z* = 2.29, *p* = 0.011. Our results suggest that pivotal frontal areas change temporally with WM subprocesses.

**Table 4:** Fit indices of models for genotype groups.

Group (task phase)	*χ* ^2^	*df*	*p*	AIC	CFI
COMT genotypes (encoding)					
MM/VM	5.12	2	0.077	29.12	0.994
VV	79.75	2	0.001	53.75	0.826
COMT genotypes (delay)					
MM/VM	59.22	2	0.001	43.22	0.921
VV	88.93	2	0.001	70.93	0.748
HTR2A genotypes (encoding)					
AA/GA	80.36	2	0.001	54.34	0.802
GG	62.35	2	0.001	43.41	0.885
HTR2A genotypes (delay)					
AA/GA	69.41	2	0.001	48.35	0.865
GG	77.85	2	0.001	51.85	0.848

Non-significant *χ* ^2^ statistic represents a good fit to the data. A lower AIC value indicates a better fit, whereas a higher CFI value indicates a better fit. *df*, degrees of freedom; AIC, Akaike information criterion; CFI, comparative fit index.

**Fig 5:**
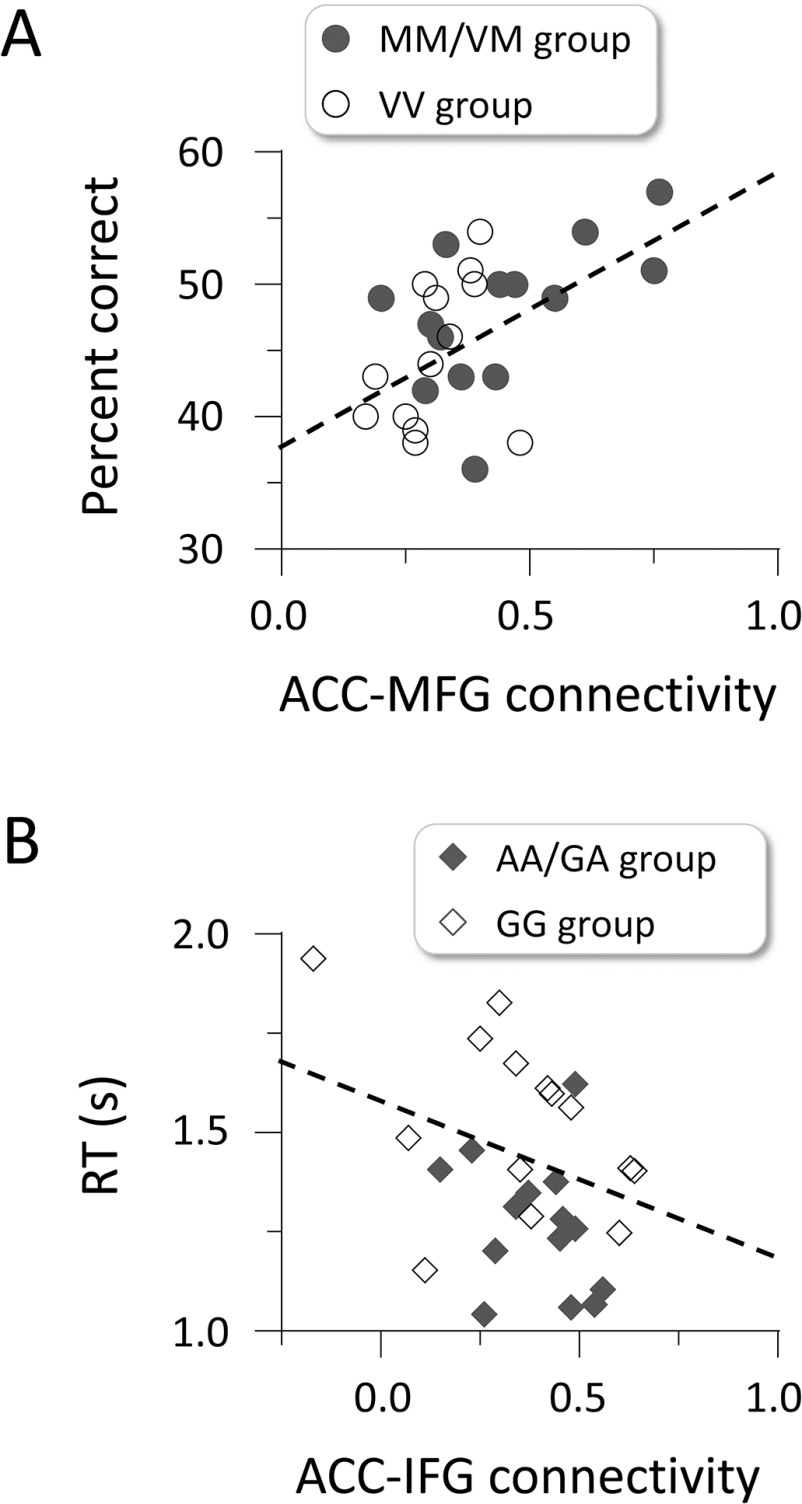
Correlations between effective connectivity and task performance. (A) Path coefficients between the ACC and MFG activations show a positive correlation with the percentage of correct responses. (B) Path coefficients between the ACC and IFG activations show a negative correlation with RTs for recognition. Circles and diamonds indicate individual data.

We checked the brain-behavior relationship to confirm the results of the connectivity analysis. For each individual, we computed the ACC-MFG connectivity during the encoding phase and the ACC-IFG connectivity during the delay phase. The former was positively correlated with recognition accuracy: *r*_(26)_ = 0.53, *p* = 0.004 (Fig 5A). The latter showed a negative correlation with RTs: *r*_(26)_ = -0.38, *p* = 0.044 (Fig 5B). These results suggest that the strong connectivity between ACC and MFG activations contributes to better recognition accuracy, whereas the strong connectivity between the ACC and IFG activations is associated with faster RTs for recognition.

## Discussion

The present results demonstrate that (1) the COMT genotypes are related to recognition accuracy, whereas the HTR2A genotypes are associated with RTs for recognition; (2) MFG and ACC/IPL activations are sensitive to differences between the COMT genotypes and between the HTR2A genotypes, respectively; and (3) strong connectivity in the cingulo-frontal networks is closely linked to better WM performance, regardless of the genotypes. Our findings suggest that the dopaminergic and serotonergic systems play different roles in the WM subprocesses and modulate closer cooperation between lateral and medial frontal activations.

Individual differences in cognitive functions, such as intelligence, personality, and social attitudes, are influenced by both genetic and environmental factors [39, 40]. We found that the COMT and HTR2A genotypes account for a 20.8% variance in WM performance. Specifically, the contribution of the COMT genotypes (effect size, *d* = 0.81 and *r* ^2^ = 0.14) identified in this study was relatively higher than that observed in previous studies that used cognitive tasks [41]. This is perhaps due to the need for executive functions to select task-relevant information and keep it as an active representation. It has been argued that visual WM tasks impose a greater demand on executive functions than verbal WM tasks because the rehearsal system for visual information is not as well-established as that for phonological information [23, 24]. In addition, executive functions are greatly influenced by genetic factors and are possibly even more heritable than intelligence [42]. Thus, it is plausible that the severely limited capacity in visual WM leads to the recruitment of executive functions and increases the effects of genetic variations on WM performance.

WM-related activations were found in the MFG, IFG, ACC, and IPL. Recent views of the parietal cortex indicate that the IPL seems to participate not only in spatial perception but also in the detection of salient items [43]. It is known that the ACC plays an important role in performance monitoring [44–46]. Neuroimaging studies using a delayed recognition paradigm have demonstrated that the MFG and IFG are responsible for the encoding and retention of information, respectively [47, 48]. In addition, WM-load-dependent activity has been found in the MFG: larger set sizes (i.e., the number of memory items) elicit greater encoding-related activity [49–51] and greater retention-related activity [52–54]. Taken collectively, these findings suggest that the MFG and IPL are engaged in creating mental images from visual inputs and preserving the images, whereas the ACC and IFG are involved in maintaining a mental set for task management and controlling distractor interferences.

Genetic factors affect individual variations in frontal and parietal activations during a WM task [55]. We showed that the COMT and HTR2A genotypes have different brain-behavior patterns. For the COMT genotypes, recognition accuracy was better in the MM/VM group than in the VV group, and the MFG for the former was less active during the encoding and delay phases. This pattern is consistent with previous findings showing that cognitive abilities are better and frontal activations are lower for Met carriers than for Val carriers [7–9]. It has been also found that brain activity decreases more for good performers than for poor performers in visual WM tasks [56–58] and verbal WM tasks [51, 59, 60]. These findings support a neural efficiency hypothesis in which the same neural computation is performed, but the metabolic expenditure differs between the groups: one group is efficient because it can perform a task at a lower metabolic cost than the other group. For the HTR2A genotypes in the present study, the RTs for recognition were faster in the AA/GA group than in the GG group, and the ACC and IPL for the former were more active during the delay phase. On the other hand, other studies have found that brain activity increases more for good performers than for poor performers in visual WM tasks [61, 62] and verbal WM tasks [63–65]. These findings support a mental effort hypothesis in which the concentration of mental resources on task requirements results in better performance. However, the neural efficiency and mental effort hypotheses appear superficially to be exclusive, pointing to the need for some other interpretation. Several researchers have attempted to account for the brain-behavior relationship in terms of the interaction between factors: neural efficiency and task difficulty [66]. However, it should be noted that although the degree of task difficulty was not manipulated in the present study, the results revealed the co-occurrence of different brain-behavior patterns within a WM task.

Importantly, we revealed that WM performance depends on the connectivity in interregional networks. First, strong connectivity between ACC and MFG activations was associated with better recognition accuracy in the MM/VM group. The ACC and MFG receive extensive projections from dopaminergic neurons in the subcortical areas [29]. Thus, it is likely that the ACC-MFG network is modulated by the dopaminergic system. Second, strong connectivity between ACC and IFG activations was linked to faster RTs for recognition in the AA group. The importance of inhibition in WM systems has been argued in terms of human behaviors [67, 68]. WM performance is predicted not only by the neural enhancement of task-relevant information but also by the neural suppression of distractors [69, 70]. In addition, an fMRI study using various cognitive tasks has demonstrated that the ACC-IFG network is involved in task-set maintenance [71]. Thus, the ACC-IFG network is probably modulated by the serotonergic system. Our findings may be consistent with the dual-network model of top-down control, in which the front-parietal network initiates and adjusts control signals and the cingulo-opercular network provides stable task-set control [72].

It is known that the allele frequencies of COMT Val/Met genotypes vary with ethnic backgrounds. Europeans have two alleles of nearly equal frequencies, whereas the Val allele is much more common in other populations [73]. The genotype distribution of the Japanese sample identified in this study (MM, VM, and VV; proportion = 0.18, 0.42, and 0.40, respectively) is very similar to that of an East Asian sample observed in a global survey (proportion = 0.24, 0.36, and 0.40, respectively) [73]. As described above, our results are consistent with activation patterns in COMT genotype groups based on a European sample [7, 9]. Taken together, the present findings as regards COMT genotypes could be generalized. However, further research on HTR2A genotypes is needed because reliable findings have yet to be accumulated.

Finally, we mention some limitations of the present study. Although we found significant differences between the behavioral and brain data of the genotypes, the number of participants (*N* = 28) was not very large. A post-hoc power analysis showed that 40 participants were needed to achieve a statistical power of 80% to detect behavioral differences between the genotype groups (*α*-level = 0.05). Meta-analysis studies have pointed out that a sample size of more than 60 or 70 participants is necessary for imaging genetics research to enhance the statistical power [74, 75]. In addition, the differences between the activation areas of the genotype groups were smaller than expected. This may be because the task paradigm used in this study was insufficient to disentangle the encoding and delay activations: the inter-trial interval was jittered but the inter-phase interval was fixed. We placed emphasis on the relationship between the cingulo-frontal networks and visual WM performance. However, several researchers have argued that the activity of the parietal areas reflects individual differences in visual WM performance [76–78]. Thus, it is important to specify the functional roles of the frontal and parietal areas in WM as well as replicate the analyses with large sample data.

In summary, it has been argued that WM supports the fundamental processes of cognitive abilities. However, there is no consensus as to how the dopaminergic and serotonergic systems influence WM subprocesses. The present study used an imaging genetics approach to examine this issue. We found that synchronized frontal activations during encoding and retention processes are affected by the dopaminergic and serotonergic systems. We believe that examining the connectivity between brain activations is a useful way to gain an integrative understanding of the relationship between neurochemical functions and human behaviors.
